# To Infection and Beyond: The Multi-Pronged Anti-Cancer Mechanisms of Oncolytic Viruses

**DOI:** 10.3390/v8020043

**Published:** 2016-02-04

**Authors:** Kevin A. Cassady, Kellie B. Haworth, Josh Jackson, James M. Markert, Timothy P. Cripe

**Affiliations:** 1 Center for Childhood Cancer and Blood Diseases, Nationwide Children’s Hospital, The Ohio State University, 700 Children’s Drive, Columbus, OH 43205, USA; kevin.cassady@nationwidechildrens.org (K.A.C.); Kellie.Haworth@nationwidechildrens.org (K.B.H.); 2 Division of Infectious Diseases, Nationwide Children’s Hospital, The Ohio State University, 700 Children’s Drive, Columbus, OH 43205, USA; 3 Division of Hematology/Oncology/Blood and Marrow Transplant, Nationwide Children’s Hospital, The Ohio State University, 700 Children’s Drive, Columbus, OH 43205, USA; 4 School of Medicine, University of Alabama-Birmingham, Birmingham, AL 35233, USA; josh.jackson.ish@gmail.com; 5 Department of Neurosurgery, University of Alabama-Birmingham, Birmingham, AL 35233, USA; jmarkert@uabmc.edu

**Keywords:** oncolytic virotherapy, immunotherapy, cancer

## Abstract

Over the past 1–2 decades we have witnessed a resurgence of efforts to therapeutically exploit the attributes of lytic viruses to infect and kill tumor cells while sparing normal cells. We now appreciate that the utility of viruses for treating cancer extends far beyond lytic cell death. Viruses are also capable of eliciting humoral and cellular innate and adaptive immune responses that may be directed not only at virus-infected cells but also at uninfected cancer cells. Here we review our current understanding of this bystander effect, and divide the mechanisms into lytic, cytokine, innate cellular, and adaptive phases. Knowing the key pathways and molecular players during virus infection in the context of the cancer microenvironment will be critical to devise strategies to maximize the therapeutic effects of oncolytic viroimmunotherapy.

Most of us who entered the field of oncolytic virotherapy over the past two decades were originally attracted by the simple idea of a lytic virus infecting and killing a tumor cell. Indeed, the possibility of harnessing the capability of lytic viruses—which have evolved over millennia to efficiently invade, subsume and destroy cells—for cancer therapy has intrigued the lay public as well, being featured in popular novels, television shows and movies [1,2,3,4,5]. The presumptive association of virus permissivity with antitumor efficacy has pervaded the field from the very beginning with laboratory experiments propagating viruses in animal tumors nearly 95 years ago [6,7] through most of the past century of research in this area [8,9,10,11,12,13]. In fact, the notion that therapeutic efficacy is directly related to the capacity for lytic infection has driven most investigators and many pharmaceutical companies to seek strategies for increasing virus replication efficiency [14,15,16]. While still important, we now know the mechanisms by which virus infections induce cancer regressions extend far beyond the simple infection of individual tumor cells.

The fact that viruses induce antigen-specific, adaptive anti-cancer immune responses has been known for several years. Early reports in the late 1990s made it clear, if not under-appreciated at the time, that viral infections of tumors in animal models could reveal and/or elicit cancer antigen-specific adaptive immune responses, functioning as *in situ* cancer vaccines [17,18]. Helping to stimulate such immunity was the basis for inserting the immunomodulatory gene for the secretion of GM-CSF into the virus now known as Talimogene Laherparepvec (T-VEC), the first oncolytic virus to be licensed by the FDA as a cancer therapeutic. Indeed, in the seminal trial that lead to its approval, 77.5% of virus-injected melanoma skin or nodal lesions decreased in size, as did 52.3% of noninjected nonvisceral lesions and 29.9% of noninjected visceral lesions, making a strong argument for an immunologic effect [19]. Recent studies suggest that even the updated concept of lytic viruses causing both direct cell killing and the induction of anti-tumor T cells under-represents the full anti-tumoral therapeutic impact of oncolytic virotherapy.

Our understanding of oncolytic virotherapy has progressed in parallel with our understanding of tumor biology. Rather than a simple clump of unstrained cancerous cells, we now appreciate that solid tumors are interconnected ecosystems comprised not just of cancer cells but also of numerous non-malignant cells, each likely playing diverse roles in enabling tumor growth and persistence. Although variable by tumor type and location, if not also each individual patient and area within a given tumor, the tumor microenvironment is often composed of stromal cells such as vascular endothelial cells, pericytes, tumor-associated fibroblasts, hematopoietic cells, innate immune cells such as macrophages, neutrophils, and myelocytes, and adaptive immune cells such as lymphocytes, each with numerous subsets and so-called polarities. Cancer cells leverage these non-cancer cells to help them grow and to evade immune detection. For example, cells may secrete TGF-β, IL-10 and prostaglandin E2, which down-regulate T lymphocyte immune recognition and cytokine production [20,21,22,23]. Regulatory T cells (T_Regs_) and tumor-associated macrophages (TAMs) within the tumor microenvironment contribute to elevated IL-10 production, which functionally impairs infiltrating T effector cells. Tumors also may express molecules that directly inhibit cytotoxic T cells, such as CTLA-4 and PD-L1 [24,25]. With this relatively new knowledge of the intricate capabilities of solid tumors to evade the immune system, we have come to also learn that viral infections of tumors likely affect many (if not all) of these cells that contribute to this immunosuppressive milieu, either directly by their infection or indirectly by the induction of immunostimulatory cytokines and chemokines [26,27,28,29]. While viral replication and the direct cytolytic phase of oncolytic virotherapy may serve as a “tumor debulking mechanism”, it may also play a role in exposing tumor neoantigens to antigen presenting cells and lead to immune-mediated anti-tumor responses.

There is now ample evidence that various anti-viral immune responses contribute to oncolytic virus anti-tumor efficacy. Virus-induced Type I interferon (IFN) signaling ultimately leads to the secretion of mutiple immunostimulatory cytokines and chemokines. In some cases, production of cytokines such as TNF-α, TRAIL, and even type I interferons themselves may have direct cytotoxic effects on neighboring, uninfected cancer cells, depending on their susceptibility [30]. Knowledge of this tumoricidal cytokine-mediated phase during virus infection has led to strategies to increase the cytotoxic effects of virotherapy by potentiating the susceptibility of cancer cells to apoptosis-inducers such as SMC mimetics [30,31]. Beug *et al.* observed the effect with several viruses including vesicular stomatitis virus, Maraba, vaccinia, HSV1, and reovirus, though it was less dramatic in those with elaborate mechanisms to suppress innate immune signaling. In addition, activation of pattern recognition receptors by pathogens such as viruses (e.g., TLR, RIG-I, MDA5, STING, IFI16) leads to adjuvant-like effects that are instrumental in stimulating immune recognition and adaptive immune memory. Induction of these receptors not only induces innate immune responses but are also important in antigen presentation and generation of robust adaptive immune responses [32,33,34]. Consequently, there has been increased interest in methods such as the use of toll-like receptor agonists that harness this response in both the vaccine and immunotherapy fields [32,35].

As intended during a normal virus infection, chemokine and cytokine production result in further recruitment and activation of innate immune cells (neutrophils, NK cells, and macrophages) and adaptive (CD4+, CD8+) T lymphocytes [21,36]. Although these events contribute to viral clearance [37,38,39,40], they are thought to transiently reverse the immunosuppressive environment and stimulate anti-tumor responses [41,42,43,44,45]. Furthermore, compared with their relevant control viruses, oncolytic viruses designed to express pro-inflammatory genes show enhanced anti-tumoral effects. For example, oHSV engineered to express IL-12, IL-18, or IL-4 has improved anti-tumor efficacy [46,47,48,49,50]. Similarly, oncolytic adenovirus co-expressing IL-12 and IL-18 enriches tumor-specific immunity via the differentiation of T cells [51] and treatment with an IL-12 and CCL2 co-expressing virus increases recruitment of activated macrophages and T cells and improved survival without decreasing viral replication [42,43,52]. Some of the effects of control and transgene-enhanced viruses correlating with increased antitumor efficacy include changes in macrophage polarization as shown with paramyxovirus and adenovirus [53,54], reduced Tregs, and changes in TGF-β and IL-10 levels [55]. Interestingly, preexisting antiviral immunity does not always diminish and occasionally enhances the antitumor efficacy of virotherapy [21,56,57]. In some cases, survival advantages seen in immunocompetant tumor models is lost in immune suppressed mice [21,58]. In other models, however, recruitment of innate immune cells rapidly clears replicating oHSV and is detrimental to oncolytic virus therapy by limiting viral replication [26]. Finally, transcriptional array analyses from a Phase Ib clinical trial of an oncolytic virus (HSV1-derived Δγ_1_ 34.5-deleted G207) suggest that anti-viral immune responses contributed to anti-cancer activity, as long-term survivors exhibited greater inflammatory and interferon-stimulated gene expression compared to non-responders [59]. In fact, there is growing evidence that in some cases, the cascade of immunologic effects may be elicited by detection of virus proteins or genomes, even in the absence of any virus replication [60,61,62].

These observations support at least four distinct but overlapping phases of oncolytic virotherapy efficacy: (1) direct cellular lysis; (2) cytokine-induced apoptosis; (3) innate immune cell cytotoxicity; and (4) antigen-specific adaptive T cell killing (Figure 1). The extent to which each phase plays a role in the regression of an individual patient’s tumor likely varies by virus species and strain, its attenuating mutation(s) (if any), the presence of any engineered transgenes within the viral genome, characteristics of the tumor cell itself (e.g., interferon responsiveness), characteristics of the tumor’s microenvironment, and the immunologic status of the patient.

**Figure 1:**
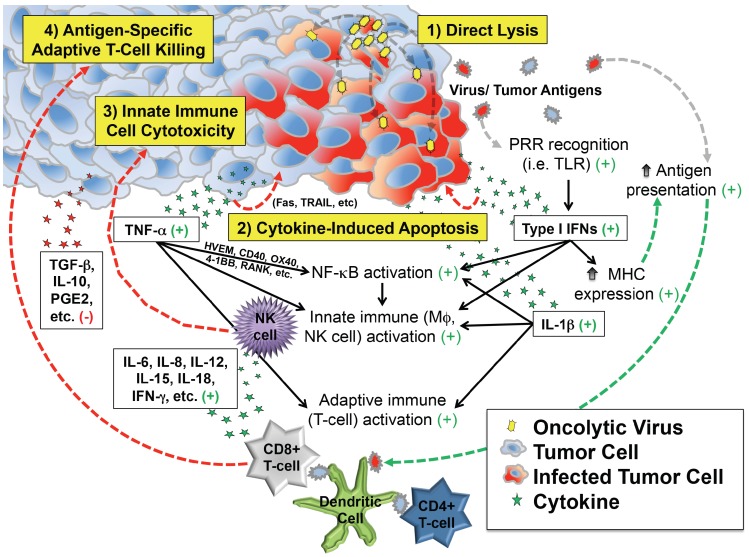
Depiction of critical events beyond infection that contribute to antitumor efficacy of virotherapy. In addition to direct lysis of tumor cells, infection induces secretion of cytokines and chemokines that can kill cancer cells directly and also recruit and activate innate and adaptive immune cells that attack the tumor. Most of the downstream effects of infection are favorable for tumor therapy (indicated by the green plus signs), which counteract immunosuppressive molecules (red minus sign) in the tumor microenvironment.

While there is a consensus that the immune response contributes to oncolytic virotherapy, there are several unanswered questions that need to be addressed. Many of the past studies have involved empiric approaches using animal models suited for a particular oncolytic virus. Animal models and even mouse strains can differ in their immunologic responses to the same virus [63,64], which in theory may affect oncolytic viral effects. Presently we do not know if differences in virus mechanisms observed are a function of the unique model systems and experimental methods used in various studies or whether these are a function of unique viral-host evolutionary pathways. This raises the question as to whether immune-mediated responses differ between various oncolytic viruses, or is there a common response necessary for successful oncolytic virotherapy? Virally-induced immune-mediated responses have many similarities [65], all of which are thought to contribute to the anti-tumor effect. As we move forward, there will be a need to develop collaborative and cooperative approaches to resolve some of the current mechanistic differences reported.

In addition to mechanistic variances between viral vectors, there are also discrepancies among tumor types. Alternate approaches may be necessary based upon the unique tumor biology involved in different cancer subtypes. For example, some tumors possess up-regulated IFN signaling pathways [66,67,68] suggesting cells are primed to resist oncolytic viruses prior to infection. These tumors have evolved to escape the IFN-mediated anti-tumor effects of this chronic stimulation, but accumulate interferon-stimulated genes that may limit initial viral infection and gene expression. Certainly oncolytic viruses derived from vesicular stomatitis virus, measles, semlikivirus forest and respiratory synticial virus are affected by the antiviral state found in some cancers [69,70,71,72,73,74]. Furthermore, a strategy involving transient immunosuppression may benefit oncolytic virotherapy in these instances such as has been shown in preclinical models for reovirus [75], though such an approach may eliminate the principal immune-based anti-tumor efficacy of an oncolytic virus for other tumor types.

Another issue is the relative immunogenicity of virus *vs.* tumor antigens. Viral antigens usually contain immunodominant epitopes that elicit strong anti-viral immune responses, which may limit the development of robust anti-tumor immunity by essentially “overshadowing” less immunodominant tumor antigens. Virus immunodominance has not been adequately examined in the context of oncolytic virotherapy, so the extent to which it may represent a barrier to tumor immunotherapy is unknown. The issue likely varies among different virus types since they encode different host evasion mechanisms, which are functional during an oncolytic virus infection (unlike non-live virus vaccines). One potential strategy to mitigate the immunodominance of virus antigens is to use a heterologous prime-boost, which is designed to train the immune response against specific tumor antigens using different virus vectors. Such an approach has been utilized successfully with sequential use of adenovirus and vesicular stomatitis virus [76].

As the field matures and more agents advance to clinical trials, it is essential that we begin to examine correlative data from clinical trial samples with different oncolytic virotherapeutics to answer several other important questions. First, what is the role of the innate immune-mediated response with virotherapy? For example, some investigators have identified that the NK cell restricts oHSV replication and anti-tumor effect in their model systems [26]. However, others [77] have identified NK cells as responsible for an adenoviral-mediated anti-tumor response. Are the differences a function of the model systems or are different unique viruses responsible for specific anti-tumor immune effectors? Further work is needed to fully understand these differences.

Early interest in the innate response focused on antiviral and anti-tumor effectors. However, innate myeloid cells associated with the tumor are important mediators of tumor immune evasion. How virotherapy directly or indirectly modifies immunosuppressive myeloid-derived cells, including macrophage polarity, in the tumor microenvironment is another area of interest, raising the next question: By engineering viruses or using adjuvants that modify these tumor-associated cells, can we regulate their functions and thus the immune response to improve virotherapy activity and provide durable anti-tumor immunity? In this regard, toll-like receptor agonists such as poly I:C and CpG oligonucleotides have a proven role in priming vaccine immunotherapy [32,35,78] and thus are of interest to test in combination with virotherapy. Also yet to be thoroughly explored in the context of virotherapy are agents that deplete or alter polarization of tumor-associated macrophages such as trabectadin [79].

With the dawn of the immunotherapy age, we have continued to advance our knowledge of how cancers effectively avoid the immune system, and apply what we know to new therapeutic strategies. This raises the next crucial question: Does immune editing and clonal escape threaten to cripple virotherapy like it has for single antigen immune-mediated approaches? A recent study showed that adenovirus infection of a lung cancer model elicited T cell responses to neoantigens and overcame resistance to T cell checkpoint blockade [80], suggesting immune escape may not be a major issue. In addition, because oncolytic virotherapy elicits multi-pronged anti-cancer mechanisms, its use may have the potential to supersede the immune evasion strategies put forth by solid tumors.

Lastly, what role does viral replication or viral antigen production play in eliciting the immune-mediated anti-tumor response? Does triggering intrinsic antiviral response pathways serve as a necessary adjuvant for adaptive immunity against the tumor while additionally inducing antiviral effectors that limit viral replication and cytolytic activity? If so, can this antiviral response be delayed temporarily without compromising safety (infection of normal tissues) or interrupting the anti-tumoral immune-mediated effects to improve overall tumor response? For example, one approach might be to induce immunosuppression early after virotherapy to enable virus replication and make it only transient, or to utilize immunosuppression only during the first few treatments but omit it during later virus injections.

As our understanding of tumor biology and the effects of virus infection within the complex tumor microenvironment continues to evolve, we are likely to reveal even more varied mechanisms underlying virus-induced cancer regressions. It would be precarious to rush to a single mechanism that explains effective oncolytic virus therapy while neglecting other established mechanisms. With growing interest in dissecting the immune-mediated virotherapy anti-tumor response, we run the risk of discarding viral replication as an important virotherapy function. Effective virotherapy is likely multifactorial and involves both direct and indirect components. Knowing these mechanisms should enable the design of rational strategies to leverage and augment each virally-induced phase with the sequential use of adjuvant small molecule and other biologic therapeutics resulting in maximal anti-tumor impact. Part of the challenge will be to also identify predictive biomarkers of each therapeutic phase, so we can further personalize oncolytic virotherapy by determining which enhancements will be most effective for a given patient.
